# Current Landscape of Chronic Inflammatory Dermatoses: Where We Are and Where We Are Heading

**DOI:** 10.3390/cimb46090611

**Published:** 2024-09-16

**Authors:** Aleksandra Sójka, Piotr K. Krajewski

**Affiliations:** University Centre of General Dermatology and Oncodermatology, Wroclaw Medical University, 50-556 Wrocław, Poland; aleksandraa.sojka@gmail.com

## 1. Introduction

Chronic inflammatory dermatoses represent a heterogeneous group of skin disorders that are often characterized by persistent and relapsing inflammation, with complex underlying pathomechanisms. These disorders, which include, e.g., hidradenitis suppurativa (HS), psoriasis, atopic dermatitis (AD), and alopecia areata (AA), are driven by dysregulated immune responses, including Th1 and Th2 pathways [1]. Despite the diversity of these conditions, they share common features such as chronicity, substantial morbidity, and a significant impact on patients’ quality of life. As we launch this Special Issue of *Current Issues in Molecular Biology* focusing on chronic dermatoses, it is important to examine the current landscape of treatment strategies, from traditional systemic therapies to novel biologics, and to explore future directions in managing these complex conditions.

## 2. Traditional Systemic Therapies: Limitations and Challenges

For decades, the management of chronic inflammatory dermatoses has relied on the use of traditional systemic immunosuppressive and immunomodulatory drugs. Agents such as methotrexate (MTX), cyclosporine A, dapsone, and glucocorticosteroids (GCs) have been the mainstay of therapy, providing symptomatic relief for many patients. These medications have broad immunosuppressive effects, which can be beneficial in controlling inflammation. For instance, MTX, a folate antagonist, inhibits dihydrofolate reductase and reduces the proliferation of immune cells and keratinocytes, making it a successful therapy for psoriasis [2]. Among patients receiving MTX for 12–16 weeks, 60–70% showed a 75% improvement in the Psoriasis Area and Severity Index (PASI 75) [2]. Similarly, cyclosporine A, a calcineurin inhibitor, suppresses T-cell activation and cytokine production, leading to rapid disease control. It has effectively treated moderate-to-severe cases of AD and psoriasis [3,4]. GCs remain a mainstay in managing various chronic inflammatory dermatoses, including psoriasis, atopic dermatitis, and atopic dermatitis. Their broad-spectrum anti-inflammatory effects result in rapid symptom relief, making them indispensable for acute flares.

Nevertheless, it should be emphasized that despite their widespread use, these medications are not free of side effects. Due to their non-specific nature, they can suppress the immune system, leading to an increased risk of infections and malignancies. Moreover, their efficacy can be variable, with many patients experiencing partial responses or treatment failure. In addition, the chronic nature of these diseases often requires long-term therapy, which can lead to cumulative toxicity. For instance, the prolonged use of cyclosporine A is associated with nephrotoxicity and hypertension, while MTX can lead to hepatotoxicity and bone marrow suppression [5,6]. While highly effective in the short term, GCs are known for their systemic side effects, including osteoporosis, hyperglycemia, and adrenal suppression, when used chronically [7]. As a result, there has been a growing need for more targeted therapies that can offer better efficacy with a more favorable safety profile.

## 3. The Advent of Biologics: A Paradigm Shift in Treatment

The introduction of biological therapies has revolutionized the management of chronic inflammatory dermatoses, offering a more targeted approach to treatment. Biologics are humanized or human monoclonal antibodies or fusion proteins designed to specifically target key molecules involved in the pathogenesis of chronic inflammatory dermatoses, allowing for more effective disease control with a reduced risk of side effects.

The first biologic approved by the U.S. Food and Drug Administration (FDA) in 1998 for dermatological treatment was etanercept, a TNF-α inhibitor monoclonal antibody for psoriasis [8]. This initiated a new phase in dermatology, where treatment could be customized to target specific immune pathways. Since then, the repertoire of biologics has expanded significantly, offering targeted treatment options that modulate particular components of the immune response [9].

In psoriasis, the efficacy of TNF-α inhibitors (etanercept, infliximab, and adalimumab) is well established, providing rapid and sustained clearance of psoriatic lesions [10]. Ustekinumab, an IL-12/23 inhibitor, further advanced the treatment options by offering an alternative mechanism of action with a favorable safety profile. More recently, IL-17 inhibitors (secukinumab, ixekizumab, and bimekizumab) and IL-23 inhibitors (guselkumab and tildrakizumab) have demonstrated even greater efficacy, with some patients achieving complete or near-complete skin clearance [11].

The approval of dupilumab, an IL-4 receptor alpha antagonist, has marked a significant breakthrough in the treatment of AD [12]. Dupilumab blocks the signaling of both IL-4 and IL-13, which are cytokines with a crucial role in the Th2-mediated immune response characteristic of AD [13]. Additionally, clinical trials and real-world data have shown that dupilumab significantly improves skin clearance and reduces pruritus, with a safety profile superior to traditional systemic therapies [13,14]. Other biologics targeting IL-13 inhibitors (tralokinumab and lebrikizumab) have been recently approved for use in adult patients with moderate-to-severe AD. Clinical trials showed a significant decrease in AD and pruritus severity, as well as an important improvement in patients’ QoL [15].

Hidradenitis suppurativa, a condition notoriously resistant to treatment, saw the approval of adalimumab, a TNF-α inhibitor, as the first FDA-approved biologic for moderate-to-severe cases in 2015 [13,14]. While adalimumab provides relief for many patients, its efficacy is variable, and not all patients achieve significant improvement [16,17]. The FDA’s approval of bimekizumab, a dual IL-17A and IL-17F inhibitor, and secukinumab, an IL-17A inhibitor, has marked a significant milestone in the treatment of HS [18,19]. Both agents have demonstrated promising results in clinical trials, offering a new level of efficacy in reducing the inflammatory lesions and pain associated with HS. These approvals underscore the growing understanding of the pivotal role of IL-17 in the pathogenesis of this disease and represent a significant step forward in providing more effective and targeted treatment options for patients [18,19].

Alopecia areata, traditionally managed with corticosteroids, has seen limited success with biologics. Another significant development was the introduction of Janus kinase (JAK) inhibitors for the treatment of AA. Tofacitinib, the first JAK inhibitor approved by the FDA for dermatological use, has shown promise in treating moderate-to-severe AA [20]. Subsequently, other JAK inhibitors, including baricitinib, ruxolitinib, and ritlecitinib, have been approved for the treatment of moderate-to-severe AA [21].

**Figure 1:**
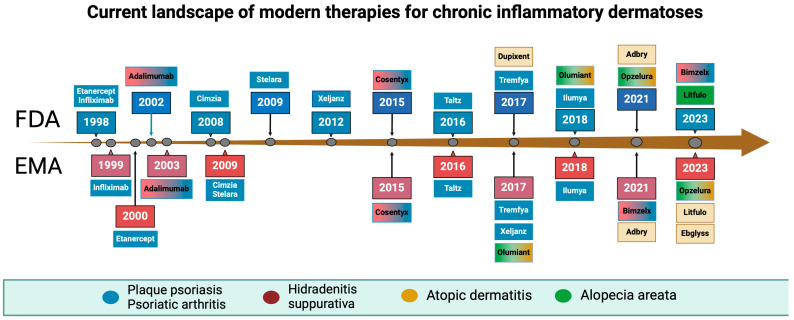
Timeline of biological drugs and Janus kinase inhibitors acceptance by the Food and Drug Administration and the European Medical Agency. Created with BioRender.com.

JAK inhibitors have also shown efficacy in other chronic inflammatory dermatoses. Baricitinib, for example, is approved for the treatment of moderate-to-severe atopic dermatitis, offering another targeted option for patients. The potential of JAK inhibitors extends beyond current indications, with ongoing research exploring their use in psoriasis and HA [22]. The timeline of FDA and EMA acceptance of new drugs in dermatology is illustrated in Figure 1.

The exact dates and indications for those drugs are mentioned in Table 1.

## 4. Future Directions: Towards Precision Medicine in Dermatology

As we look to the future of chronic inflammatory dermatosis management, the emphasis is moving more towards targeted treatments. Identifying biomarkers that can predict disease severity, treatment effectiveness, and long-term outcomes will be crucial in guiding treatment strategies. The goal is to tailor treatment based on individual patient profiles, which could include genetic, immunological, and environmental factors.

Ongoing research on the molecular mechanisms involved in the pathophysiology of these diseases may yield new therapeutic targets. For instance, the exploration of the microbiome’s role in skin disease could lead to novel interventions aimed at modulating the skin’s microbial environment. Additionally, advances in gene therapy and the use of biologics targeting novel pathways, such as IL-17, IL-23, and IL-31, will continue to expand the therapeutic options.

Long-term studies should also refine the safety and efficacy of existing biologics and JAK inhibitors, providing insights into optimal dosing regimens and combination therapies. As these treatments become more widely used, it is also essential to monitor rare side effects and develop strategies to deal with treatment resistance that may occur with long-term use.

In conclusion, the treatment landscape for chronic inflammatory dermatoses has significantly evolved over the past two decades, with biologics and JAK inhibitors providing new hope for patients who previously had limited options. As we continue to unravel the molecular foundations of these complex diseases, the future of dermatological treatment promises to be increasingly personalized, with the potential to improve outcomes and quality of life for patients worldwide.

## Figures and Tables

**Table 1:** Biologics and Janus kinase inhibitors (JAKi) that are currently available for treating chronic inflammatory dermatoses.

Drug Name	Product Name	Mechanism	FDA Approval Year	EMA Approval Year	Indications
Etanercept	Enbrel	TNF-α inhibitor	1998	2000	Plaque psoriasis, Psoriatic arthritis
Infliximab	Remicade	TNF-α inhibitor	1998	1999	Plaque psoriasis, Psoriatic arthritis
Adalimumab	Humira	TNF-α inhibitor	2002	2003	Plaque psoriasis, Psoriatic arthritis, Hidradenitis suppurativa
Certolizumab Pegol	Cimzia	TNF-α inhibitor	2008	2009	Plaque psoriasis, Psoriatic arthritis,
Ustekinumab	Stelara	IL-12/23 inhibitor	2009	2009	Plaque psoriasis, Psoriatic arthritis
Guselkumab	Tremfya	IL-23 inhibitor	2017	2017	Plaque psoriasis, Psoriatic arthritis
Tildrakizumab	Ilumya	IL-23 inhibitor	2018	2018	Plaque psoriasis
Secukinumab	Cosentyx	IL-17A inhibitor	2015	2015	Plaque psoriasis, Psoriatic arthritis, Hidradenitis suppurativa
Ixekizumab	Taltz	IL-17A inhibitor	2016	2016	Plaque psoriasis, Psoriatic arthritis
Bimekizumab	Bimzelx	IL-17A/F inhibitor	2023	2021	Plaque psoriasis, Psoriatic arthritis, Hidradenitis suppurativa
Dupilumab	Dupixent	IL-4/13 inhibitor	2017	2017	Atopic dermatitis, Prurigo nodularis
Tralokinumab	Adbry	IL-13 inhibitor	2021	2021	Atopic dermatitis
Lebrikizumab	Ebglyss	IL-13 inhibitor	Submitted	2023	Atopic dermatitis
Tofacitinib	Xeljanz	JAK inhibitor	2012	2017	Psoriatic arthritis
Ruxolitinib	Opzelura	JAK inhibitor	2021	2023	Atopic dermatitis, Vitiligo, Alopecia areata
Baricitinib	Olumiant	JAK inhibitor	2018	2017	Atopic dermatitis, Alopecia areata
Ritlecitinib	Litfulo	JAK inhibitor	2023	2023	Alopecia areata

Although the majority of adverse events reported with the newly introduced drugs are mild and transient, their long-term safety profile must be closely monitored, particularly in terms of infection risk and potential immunogenicity.
